# Gut *Bacteroidales* and AMH/INH-B ratio predict sperm retrieval: mechanistic insights via SCFA-mediated regulation of blood-testis barrier and steroidogenesis

**DOI:** 10.3389/fcimb.2026.1777930

**Published:** 2026-05-22

**Authors:** Guanping Yao, Xiong Pan, Faju Chen, Lishou Yang, Lang Zhou, Mei Peng, Xiaosheng Yang

**Affiliations:** 1 State Key Laboratory of Discovery and Utilization of Functional Components in Traditional Chinese Medicine & School of Pharmaceutical Sciences, Guizhou Medical University, Guiyang, China; 2 Natural Products Research Center of Guizhou Province, Guiyang, China; 3 Key Laboratory of Cell Engineering of Guizhou Province, Affiliated Hospital of Zunyi Medical University, Zunyi, China

**Keywords:** anti-Müllerian hormone, fecal microbiota transplantation, FSH-normal non-obstructive azoospermia, gut microbiota abundance, inhibin-B

## Abstract

**Objective:**

To establish a non-invasive predictive model for microdissection testicular sperm extraction (micro-TESE) outcomes in FSH-normal non-obstructive azoospermia (NOA) patients by integrating gut microbiota profiling with serum biomarkers.

**Methods:**

We conducted a retrospective clinical analysis of 58 men and established a busulfan-induced FSH-normal NOA mouse model. Serum hormone levels (FSH, INH-B, AMH, testosterone) were measured by ELISA, and gut microbiota was analyzed via 16S rRNA sequencing. Testicular histology and ultrastructure were assessed by H&E staining and TEM, while protein expression was evaluated by IHC, IF, and Western blot. Receiver operating characteristic (ROC) curve analysis was used to evaluate the predictive efficacy of the serum AMH/INH-B ratio for sperm retrieval outcomes.

**Results:**

In both patients and model mice, serum INH-B, AMH, and the AMH/INH-B ratio were significantly decreased (*P* < 0.01), correlating with severe spermatogenic impairment. Mice exhibited a marked reduction in the abundance of *Bacteroidales* and *Muribaculaceae*. Fecal microbiota transplantation (FMT) restored these microbial populations, improved testicular function, and upregulated key proteins involved in proliferation (PCNA, PGK2), blood-testis barrier integrity (ZO-1, Claudin11), and steroidogenesis (StAR, CYP17A1) (*P* < 0.05). Mechanistically, FMT increased serum short-chain fatty acid (SCFA) levels, which served as the chemical messengers correlating directly with the recovery of BTB proteins and steroidogenic enzymes. Clinically, the serum AMH/INH-B ratio showed strong predictive efficacy for micro-TESE outcomes, with an area under the ROC curve (AUC) of 0.92 (95% CI: 0.86-0.98), optimal cut-off value of 0.65, sensitivity of 88.2%, and specificity of 85.7%. The gut *Bacteroidales* abundance (from mouse data) was mechanistically linked to spermatogenic function, suggesting its potential as a future clinical biomarker pending validation.

**Conclusions:**

Our findings elucidate an SCFA-mediated gut-testis axis, highlighting the therapeutic potential of microbiota modulation and providing a novel tool to guide clinical decision-making, potentially reducing unnecessary surgeries in FSH-normal NOA.Additionally, the serum AMH/INH-B ratio serves as a robust non-invasive biomarker for predicting micro-TESE outcomes in FSH-normal NOA, while gut Bacteroidales abundance may represent a complementary mechanistic target for future clinical investigation.

## 1 Introduction

Azoospermia, defined as the absence of sperm in multiple ejaculates, affects approximately 1% of the male population and accounts for 10% to 15% of infertility cases (Hubbard et al., 2025; Wang et al., 2023). It is classified into obstructive azoospermia (OA) and non-obstructive azoospermia (NOA), with NOA comprising about 60% of cases (Klami et al., 2024; Shah et al., 2023). While sperm retrieval rates in OA approach 100%, the success rate of microdissection testicular sperm extraction (micro-TESE) in NOA patients is only around 50% (Ozer et al., 2024; DriuChina et al., 2023). Currently, predictive methods such as vasography or testicular biopsy are invasive, underscoring the need for non-invasive predictors. Serum reproductive hormone levels combined with gut microbiome profiling represent a promising non-invasive alternative that could avoid procedural risks and reduce psychological and financial burdens on patients (Borzan et al., 2023; Bazzar et al., 2026). However, for NOA patients with normal serum FSH levels, reliable non-invasive prediction remains challenging. While serum INH-B or AMH alone have been explored as potential predictors, their individual predictive sensitivity and specificity are often insufficient for clinical decision-making in this specific population, as they only partially reflect the complex testicular microenvironment. This underscores a critical research gap: the lack of a multi-dimensional model that integrates complementary serum biomarkers with gut microbial alterations to provide a more holistic and accurate prediction of micro-TESE outcomes. Identifying such non-invasive predictive markers is therefore of significant clinical importance.

Conventional INH-B and FSH are routinely applied to predict micro-TESE outcomes; elevated FSH correlates inversely with INH-B and predicts poor sperm retrieval in azoospermia. However, most affected patients present with normal FSH, limiting the reliability of these hormonal markers alone.Emerging evidence links gut microbiota to male infertility, with recent studies confirming that microbial disturbances can impair sperm quality through inflammatory responses and metabolic disorders (Kaltsas et al., 2024; Cannarella et al., 2024). These findings suggest that gut microbiota may regulate testicular function via a gut–testis axis, though the underlying mechanisms remain unclear. This study addressed this gap by identifying microbial signatures in a preclinical model to establish an improved non-invasive predictive framework for microTESE, while clarifying how gut dysbiosis contributes to spermatogenic dysfunction.Currently, both domestic and international research has concentrated on identifying serum reproductive hormone levels, with comparatively fewer studies examining the combined analysis of serum reproductive hormone levels and the relative abundance of specific gut microbiota. In cases of NOA, particularly when follicle-stimulating hormone (FSH) and luteinizing hormone (LH) levels are within the normal range, it remains unclear whether the status of testicular spermatogenesis can be predicted by integrating serum reproductive hormone levels with the relative abundance of specific gut microbiota (Kaltsas et al., 2025; Cavallini et al., 2021), Furthermore, the serum AMH/INH−B ratio has been recognized as a promising indicator for assessing spermatogenic function (Kong et al., 2021). Despite these advances, key mechanistic and translational gaps persist. The specific role of microbiota-derived metabolites, particularly short-chain fatty acids (SCFAs), in coordinately regulating blood-testis barrier (BTB) integrity and steroidogenic hormone balance remains unclear. Moreover, the association between the AMH/INH−B ratio and SCFAs, as well as its clinical utility as a non-invasive predictor linked to gut microbiota, has not been established.

To address this limitation, a non-invasive prediction model combining the serum AMH/INH-B ratio with the abundance of gut microbiota was developed, and its accuracy was verified in the busulfan-induced mouse model of NOA with normal FSH levels. Meanwhile, fecal microbiota transplantation (FMT) was performed and demonstrated recovery of spermatogenic function in mice, indicating the therapeutic potential of modulating gut microbiota. Collectively, this study identifies a potential biomarker strategy that significantly enhances clinical decision-making for FSH-normal NOA patients by enabling more accurate and personalized prediction of micro-TESE outcomes. This strategy could eventually reduce the need for unnecessary surgical procedures while improving the planning of sperm retrieval efforts.

## 2 Materials and methods

### 2.1 Clinical study

#### 2.1.1 Study population and ethical approval

A total of 58 male infertility patients who visited the Reproductive Center of the Affiliated Hospital of Zunyi Medical University between 2021 and 2024 were included in the study. All patients signed an informed consent form before surgery, and this study was approved by the Medical Ethics Committee of the Affiliated Hospital of Zunyi Medical University (NO: 2021-1-119).

All subjects underwent venous blood collection on an empty stomach in the early morning after a period of abstinence ranging from 2 to 7 days. Semen was separated by centrifugation at 2000×g for 10 minutes at 4 °C and stored at -20 °C for subsequent testing. Semen was collected by masturbation into sterile containers, and a routine semen quality analysis was performed according to the WHO laboratory manual for the examination and processing of human semen (6th edition). The patients were divided into two groups: the presumed fertile control group comprised 24 cases, aged between 23–45 years (mean 31.12 ± 5.44 years), who were classified as fertile based solely on normal semen parameters without confirmed clinical pregnancy, and the NOA group comprised 34 cases, aged between 24–48 years (mean 31.14 ± 5.63 years). Based on the outcomes of micro-TESE, the 34 NOA patients were retrospectively categorized post-operatively into two subgroups: the sperm retrieval positive (SRP, n=24) group and the sperm retrieval negative (SRN, n=10) group. This classification was performed retrospectively after the surgical outcomes were known to avoid any misinterpretation of prospective grouping. Notably, the control group was defined as presumed fertile only according to standard semen characteristics, which represents an important limitation given that normozoospermia does not necessarily equate to proven fertility in a clinical context.

Inclusion criteria for the NOA group: ① At least three semen quality analyses indicating azoospermia; ② Serum FSH levels within the normal range (0.7~11.1 mIU/mL); ③ Normal bilateral testicular volume size (12~25 ml); ④ G-banding karyotype analysis showing normal (46, XY). Inclusion criteria for the normal control group: ① Semen volume ≥ 1.5ml, semen pH ≥ 7.2; ② Sperm density ≥ 15×10^6/ml; ③ Total sperm count ≥ 39×10^6/ejaculate; ④ Sperm motility (%) A grade ≥ 32% or A+B grade ≥ 40%; ⑤ Sperm viability rate ≥ 58%; ⑥ Normal morphology sperm ≥ 4%. All cases with abnormal G-banding karyotype chromosomal patterns were excluded. Exclusion criteria for the NOA group: ① re-productive system diseases; ② chronic systemic diseases; ③ genetic abnormalities; ④ unhealthy lifestyle habits (such as smoking or drinking alcohol); ⑤ exposure to harmful environments; ⑥ recent medication or surgery; ⑦improper sample collection; ⑧ obesity (BMI ≥ 30), etc.

#### 2.1.2 Testicular volume measurement

Physical examination of the testes was conducted at room temperature. Patients were examined in a standing position. The examination included: (1) visual inspection of testicular size, shape, and symmetry; (2) palpation to assess testicular texture, the condition of the epididymis and vas deferens, and to detect any nodules, thickening, or hardening of the epididymal tail; and (3) assessment of whether the seminal vesicles were palpable. Testicular volume was measured, with a normal range considered to be 12~25 mL for adult males.

#### 2.1.3 Semen routine quality analysis

Semen routine quality analysis was conducted utilizing a computer-aided sperm analysis system (Beijing Weili Company) to detect semen volume, total sperm count, sperm density and their motility. Furthermore, Diff-Quick staining reagent (Shenzhen BRED Biotechnology Company) was employed for sperm morphology staining and observation.

#### 2.1.4 Epididymal and testicular sperm retrieval

After administering a 2% lidocaine spermatic cord block and local anesthesia, a 10 mL syringe pre-filled with 1–2 mL of normal saline was used. Percutaneous puncture into the head of the epididymis or two punctures along the long axis of the testicle were performed for percutaneous epididymal sperm aspiration (PESA) and percutaneous testicular sperm aspiration (PTSA). Continuous negative pressure was maintained during needle withdrawal, and the collected epididymal fluid and testicular aspirates were submitted for analysis.

### 2.2 Animal study

#### 2.2.1 Animals and ethical approval

40 male C57BL/6J mice, aged 6 to 8 weeks, were obtained from Chongqing Tengxin Biotechnology Co. Ltd., which holds the animal production license number CXK (JING) 2021-0010 (Chongqing, China). The mice were housed in the animal facility of the State Key Laboratory of Discovery and Utilization of Functional Components in Traditional Chinese Medicine (Guizhou Provincial Natural Products Research Center), Guizhou Medical University. All animal experiments were conducted in strict accordance with international ethical guidelines and laboratory animal protection and use guidelines, with approval from the Zunyi Medical University Experimental Animal Ethics Committee (License No: 2021-2-191).

#### 2.2.2 Experimental design, grouping, and model induction

The 40 male C57BL/6J mice were randomly assigned to three experimental groups:①Control Group (n=10):Age-matched fertile male mice with normal spermatogenesis and proven baseline fertility were included. These mice received daily intraperitoneal injections of sterile saline (0.1 mL) for 17 consecutive days;②NOA Model Group (NOA, n=20): Mice received daily injections of busulfan (3 mg/kg/d, dissolved in 0.1 mL of sterile saline; Sigma-Aldrich, USA) for 17 consecutive days to induce non-obstructive azoospermia.This dosage and duration were adopted to reliably establish stable spermatogenic impairment and NOA phenotypes as previously validated.③FMT Treatment Group (NOA+FMT, n=10): Mice received busulfan injections identical to the NOA Model Group. Starting from day 21 after the first busulfan injection, these mice additionally received FMT via enema every 3 days for a total of 6 administrations.

After 40 days of treatment, the testes of the mice were harvested for histological sectioning. The absence of sperm and spermatogenic cells in the testes and epididymis indicated a successful NOA model induction. The evaluation method encompasses: ①Comparing the body weight, testicular weight, and epididymal weight; counting spermatogonia, spermatocytes, spermatids, and sperm in 10 vertical sections of seminiferous tubules and sperm in 10 vertical sections of the epididymal duct; ②The exact number and mortality rate of mice in each group were recorded and compared throughout the experiment;③Selecting the group with pathological features consistent with NOA, practical feasibility as the optimal model. During the entire 40-day experimental period, no mice died in the Control Group (0/10, mortality rate 0%). In the NOA Model Group, two mice died (2/20, mortality rate 10%). In the NOA+FMT Treatment Group, one mouse died (1/10, mortality rate 10%). The overall survival rates were 100%, 90%, and 90% in the three groups, respectively, supporting the favorable safety and practicality of this low-dose continuous busulfan-induced NOA model.

The 17-day busulfan administration protocol was chosen to induce gradual but comprehensive depletion of spermatogonial stem cells, while the 40-day experimental endpoint was selected to allow sufficient time for the full manifestation of azoospermia following the disruption of at least one complete cycle of spermatogenesis in mice (approximately 35 days). A low-dose, continuous busulfan administration protocol was employed to minimize systemic toxicity and to better mimic the heterogeneous testicular failure observed in clinical NOA patients, particularly those with normal FSH levels.

The successful establishment of the NOA model was confirmed post-mortem by histological examination and sperm count analysis. The normal reference range for mouse serum FSH was set at 0.3-2.5 mIU/mL (Ran et al., 2023). Postoperative measurements confirmed that the FSH level in the NOA model group (0.8 ± 0.3 mIU/mL) did not differ significantly from that in the control group (0.7 ± 0.2 mIU/mL; *P* > 0.05), thereby partially mimicking the core reproductive phenotypic features of FSH-normal NOA patient cohort. Consistent with the heterogeneous phenotypes observed in clinical NOA, the busulfan-induced model was anticipated to yield variability in spermatogenic impairment. Therefore, following the experimental endpoint, mice in the NOA groups were further stratified based on histological findings into NOA-Azoospermia (complete absence of sperm) and NOA-Oligospermia (residual spermatogenesis) subgroups for subsequent analyses.

#### 2.2.3 FMT intervention

Fresh morning feces (5 g) from 10 normal mice were dissolved in 10 mL of sterile saline solution. 16S rRNA sequencing of donor mouse feces confirmed significantly higher abundances of the key bacterial taxa, with *Bacteroides* at 18.2 ± 3.5% and *Muribaculaceae* at 22.1 ± 4.2%, compared to the NOA model group (*Bacteroides*: 5.3 ± 1.8%; *Muribaculaceae*: 8.7 ± 2.1%; *P* < 0.01), ensuring the quality of the microbial transplant. The mixture was then sequentially filtered through 2.00 mm, 1.00 mm, 0.50 mm, and 0.25 mm stainless steel sieves. Following filtration, the sample was centrifuged at 6, 000 g for 15 minutes. The bacterial pellets were collected, washed three times with sterile saline, resuspended in 25 mL of sterile saline, and stored at 4 °C. For FMT administration, 1 mL of bacterial suspension was delivered to each recipient mouse via enema administration every 3 days (total 6 administrations).

### 2.3 Laboratory analyses

#### 2.3.1 Hormone assays

The serum levels of FSH, INH-B, AMH, LH and T were determined by ELISA according to the manufacturer’s instructions (CSB-E06871m, CSB-E08151m, CSB-E13156m, CSB-E05101m, CSB-E05101m, Cusabio Biotech). The minimum detectable concentration for INH-B is 2 pg/ml, with a linear detection range of 3.2–800 pg/ml; the reference range for AMH is 2.04 to 19.22 ng/mL, with a threshold of AMH ≥ 1.48 ng/ml. And the normal reference ranges are as follows: FSH: 0.7~11.1 mIU/mL, LH: 0.63~11.70 mIU/mL, T: ≥ 11.5 nmol/L.

#### 2.3.2 Histology and transmission electron microscopy

Unilateral testicular tissue was paraffin-embedded, sectioned, and stained with Hematoxylin & Eosin (H&E) for light microscopy to examine the distribution of spermatogonia and various stages of spermatogenic cells within the testicular tissue. For TEM, a pre-cooled sharp blade was used to excise a 1mm×1mm× 2mm testicular tissue sample, which was then immersed in 2.5% glutaraldehyde phosphate buffer (pH 7.2) for 6 hours for fixation. The prepared samples were then processed for conventional transmission electron microscopy to observe ultrastructural changes in the seminiferous tubules and interstitial cells of the testis.

#### 2.3.3 Immunohistochemistry) and immunofluorescence

IHC: Testicular tissue sections from each group of mice were deparaffinized to water through conventional procedures and antigens were retrieved by microwave. After incubation with a 3% hydrogen peroxide solution to eliminate endogenous peroxidase, normal goat serum was used for blocking. Primary antibodies against PCNA (GB12010, Servicebio), PGK2 (13686-1-AP, proteintech), HSPA4L (BS-18082R, Bioss) (all diluted 1:200) were added and incubated overnight at 4°C; after washing with phosphate-buffered saline (PBS), sections were incubated with corresponding secondary antibodies (diluted 1:200) at room temperature for 1 hour. After further washing, DAB staining and hematoxylin counterstaining were performed and neutral gum was used for mounting. For quantitative analysis, five random fields of view were captured from each slide. The percentage of the positively stained area (brown-yellow) was quantified using ImageJ software to represent protein expression levels.

IF: Paraffin sections were dewaxed to water and the tissue sections were placed in a full container of citric acid antigen retrieval solution and microwaved (medium heat for 8 minutes, stop for 8 minutes, then medium-low heat for 7 minutes) for anti-gen retrieval. A hydrophobic barrier was drawn around the tissue with a histological pen and the tissue was perforated with 0.1% Tween-20 for 20 minutes, blocked with 3% BSA for 30 minutes, and incubated with primary antibodies against SYCP3 (DF2579, Affinity), WT1 (DF6331, Affinity) and CYP11A1 (DF4697, Affinity) (all diluted 1:200), as well as proteins ZO-1 (AF5364, Affinity), SOX-9 (AF6330, Affinity) and Claudin11 (AF5145, Affinity) (all diluted 1:200) in the mouse blood testis barrier (BTB) overnight at 4 °C. The slides were then incubated with secondary antibodies iF555-Tyramide, FITC-labeled donkey anti-rabbit IgG, and CY5-labeled goat anti-rabbit IgG for 2 hours at room temperature, followed by DAPI nuclear counterstaining, resin mounting and observation under a micro-scope.

#### 2.3.4 Western blotting

The testicular tissues of five mice were lysed in RIPA lysis buffer containing protease and phosphatase inhibitors(n=5).Total protein was extracted and protein concentration was determined via BCA assay. The protein was denatured by heating in boiling water for 10 minutes and separated via sodium dodecyl sulfate-polyacrylamide gel electrophoresis, subsequently transferred to a polyvinylidene fluoride membrane. Then the membrane was blocked with 5% non-fat milk for 2 hours; incubated with primary antibodies against 3β-HSD (GB112714, Servicebio), StAR (GB111430, Servicebio), CYP17A1 (GB112095, Servicebio) and β-actin (GB15003, Servicebio) (all diluted 1:10000) overnight at 4 °C. Then the membrane was incubated with corresponding secondary antibodies (diluted 1:5000) for another 1 hour at room temperature, and developed with DAB chromogenic solution for 1–2 minutes for imaging. β-actin was used as an internal reference, the expression levels of target protein were analyzed by gray value ratio using Image J software.

#### 2.3.5 16S rRNA gene sequencing and bioinformatic analysis

Mouse feces were collected and DNA was extracted with the MN NucleoSpin 96 Soil Kit (DP812, TIANGEN) according to the manufacturer’s instructions. Purified DNA was amplified with adapter-ligated primers, and libraries were sequenced on Illumina HiSeq 6000 covering the V3-V4 region of the 16S rDNA gene.

High-quality reads were clustered into Operational Taxonomic Units (OTUs) at 97% similarity threshold using USEARCH v10.0 and classified against the SILVA 138.1 database via the Naive Bayes classifier in QIIME2 (70% confidence threshold). Alpha diversity (species complexity per sample) was calculated using QIIME2. Beta diversity was assessed via Principal Coordinate Analysis (PCoA) based on Bray-Curtis dissimilarity.

#### 2.3.6 Metabolomics analysis

Serum metabolomic profiling was conducted using a UHPLC-Q Exactive HF-X system (Thermo Fisher). Following protein precipitation with pre-cooled methanol/acetonitrile and centrifugation, the supernatant was lyophilized, reconstituted, and filtered for analysis. Chromatographic separation was performed on an Accucore C30 column (100 mm×2.1 mm, 2.6 μm) with a gradient elution program. Mass spectrometry detection operated in both positive and negative ionization modes under data-dependent acquisition. Raw data were processed using LipidSearch software for peak alignment and compound identification, followed by normalization and log10 transformation. Differential metabolites were screened based on variable importance in projection (VIP) > 1.0 from the OPLS-DA model and P < 0.05, with subsequent KEGG pathway enrichment analysis to identify significantly altered metabolic pathways.

### 2.4 Statistical analysis

The statistical analysis was conducted by GraphPad software (version 9.5.1; La Jolla, CA, USA). All the data were displayed as mean ± standard deviation (SD). For 2 group comparisons, the Student t-test was utilized. For multiple comparisons, one-way ANOVA was performed. The comparison of percentages was performed using the chi-square test. Receiver operating characteristic (ROC) curve analysis was employed to evaluate the predictive efficacy of the serum AMH/INH-B Ratio, *Bacteroidales* Abundance, and their combination for NOA. A *P* value < 0.05 was considered statistically significant.

## 3 Results

### 3.1 The predictive value of serum biomarkers for NOA patients with normal FSH

**Figure 1:**
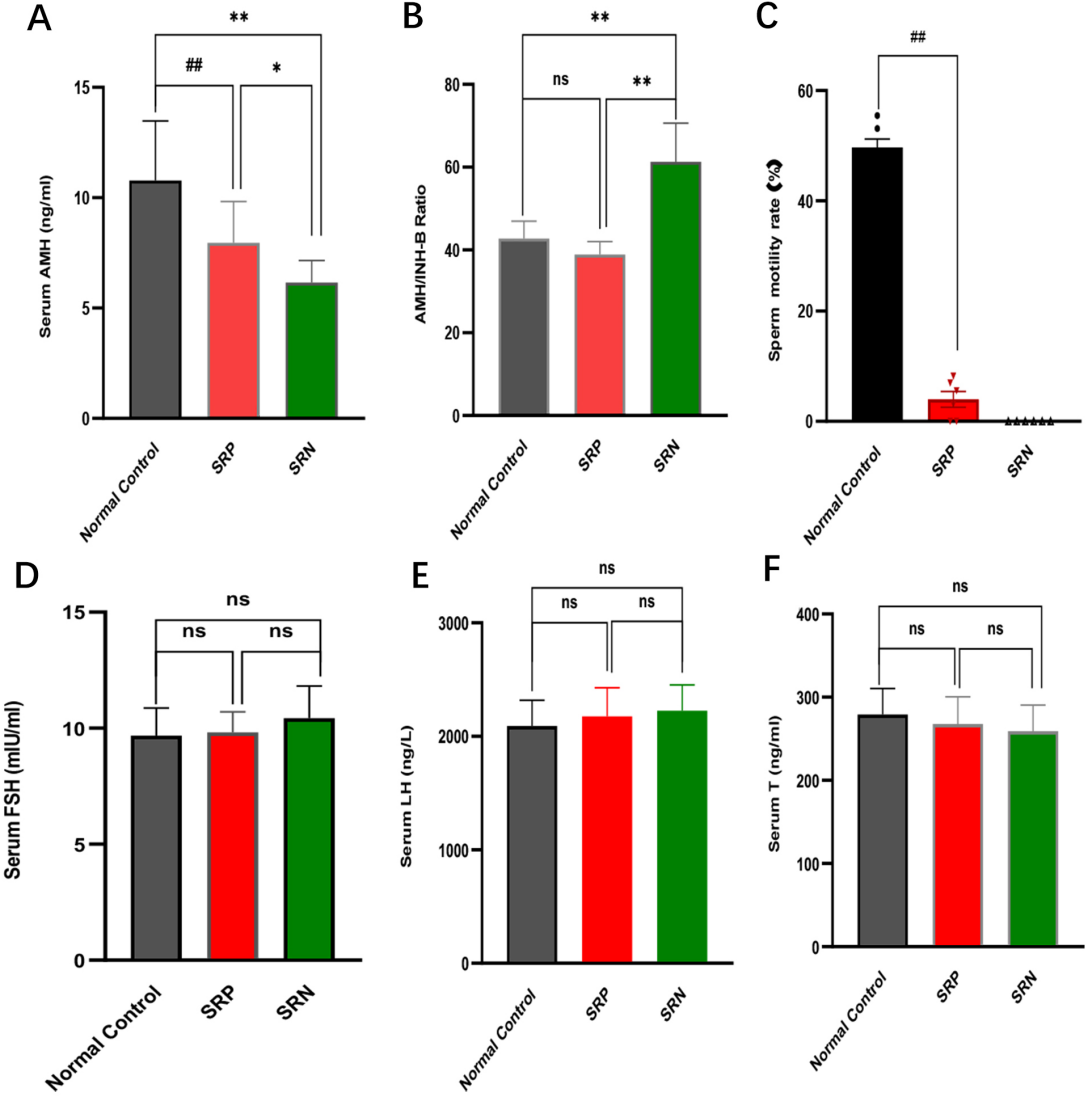
Patient baseline information. **(A)** Serum AMH levels of patients in normal control, SRP and SRN group. **(B)** AMH/INH-B ratio of patients in normal control, SRP and SRN group. **(C)** Sperm motility of patients in normal control, SRP and SRN group. **(D)** Serum FSH levels of patients in normal control, SRP and SRN group. **(E)** Serum LH levels of patients in normal control, SRP and SRN group. **(F)** Serum T levels of patients in normal control, SRP and SRN group. SRP, sperm retrieval positive group (n=24); SRN, sperm retrieval negative group (n=10). Data are presented as the mean ± SD. Statistical significance was determined using one-way ANOVA. ns, not significant. Compared with the normal control group, # *P* < 0.05, ## *P* < 0.01; Compared with the SRN group, * *P* < 0.05, ** *P* < 0.01.

This study enrolled a total of 34 patients diagnosed with NOA and normal FSH levels, along with 24 healthy controls. All patients had FSH levels within the normal range (5.16 ± 2.34 mIU/mL) (Schwarzkopf et al., 2025; Qi et al., 2021; Zheng et al., 2024), and karyotype analysis revealed a normal 46, XY chromosomal pattern (**Supplementary Figure 1**). As expected, no statistically significant differences were observed in serum levels of FSH, LH, and testosterone (T) between the NOA patient group and the healthy control group (**Figures 1D–F**).

In contrast, biomarkers directly reflecting Sertoli cell function were significantly altered. Serum AMH and INH-B levels were markedly lower in the NOA group compared to controls (AMH: 7.12 vs. 10.78 ng/mL; INH-B: 157.51 vs. 260.71 pg/mL; *P* < 0.01; **Table 1**, **Figure 1A**). When patients were stratified by micro-TESE outcomes, the sperm retrieval negative (SRN) subgroup exhibited significantly lower AMH and INH-B levels than the sperm retrieval positive (SRP) subgroup (*P* < 0.05; **Table 1**, **Figure 1A**). Consistently, the AMH/INH-B ratio was also significantly reduced in the SRN group compared to both the SRP and control groups (*P* < 0.01; **Table 1**, **Figure 1B**). Furthermore, sperm motility was significantly lower in NOA patients than in controls (*P* < 0.01; **Figure 1C**), supporting the intrinsic link between these biomarkers and spermatogenic function.

**Table 1:** Clinical characteristics and hormonal profiles of study subjects.

Group	Proportion	NOA group(n=34)	SRP (n=24)	SRN(n=10)	Normal control group (n=24)	Statistic, *p* value
INH-B(pg/ml)		157.51 ± 77.55 **^##^**	184.15 ± 62.44 *	98.80 ± 25.02	260.71 ± 72.82	*P < 0.05
INH-B ≤ 150(pg/ml)	Number of people	16	7	9	0	χ^2^ = 15.60, **^#^***P* < 0.01
	Percentage (%)	47.06	29.17	90	0	
200 <INH-B <150(pg/ml)	Number of people	6	9	1	4	χ^2^ = 0.01, **^#^***P*>0.05
	Percentage (%)	17.65	37.5	10	16.67	
INH-B ≥ 200(pg/ml)	Number of people	12	8	0	20	χ^2^ = 13.13, **^#^***P* < 0.01
	Percentage (%)	35.29	33.3	0	83.33	
PESA	Number of individuals who obtained sperm (persons)	0	24	10	N/A	
	Percentage (%)	0	70.59	29.41	N/A	
PTSA	Number of individuals without sperm (persons)	0	6	4	N/A	
	Percentage (%)	0	17.65	11.76	N/A	

PESA was conducted before PTSA, Compared with the normal control group, **^#^** *P* < 0.05, **^##^** *P* < 0.01; Compared with the SRN group, * *P* < 0.05, ** *P* < 0.01.

### 3.2 FMT improved spermatogenic disorders and hormonal imbalances in NOA mice

To validate the clinical findings, an FSH-normal NOA mouse model was established using busulfan. The busulfan treatment induced severe spermatogenic impairment with a quantifiable reduction in spermatogenic capacity, as evidenced by subsequent histological and functional analyses. As anticipated by our study design and consistent with the variability observed in clinical NOA, the busulfan-treated mice exhibited a heterogeneous phenotype, which was objectively stratified based on post-mortem histological examination and precise sperm count quantification. Specifically, the NOA model mice were divided into two subgroups: the NOA-Azoospermia group, defined by a complete absence of sperm in the epididymis (sperm count=0×10^6^/mL), and the NOA-Oligospermia group, characterized by residual spermatogenesis with a significantly reduced sperm count (0 < sperm count < 5×10^6^/mL, consistent with the clinical definition of oligospermia). This heterogeneity closely mirrors the variability seen in clinical NOA patients, confirming the validity of the established mouse model.

**Figure 2:**
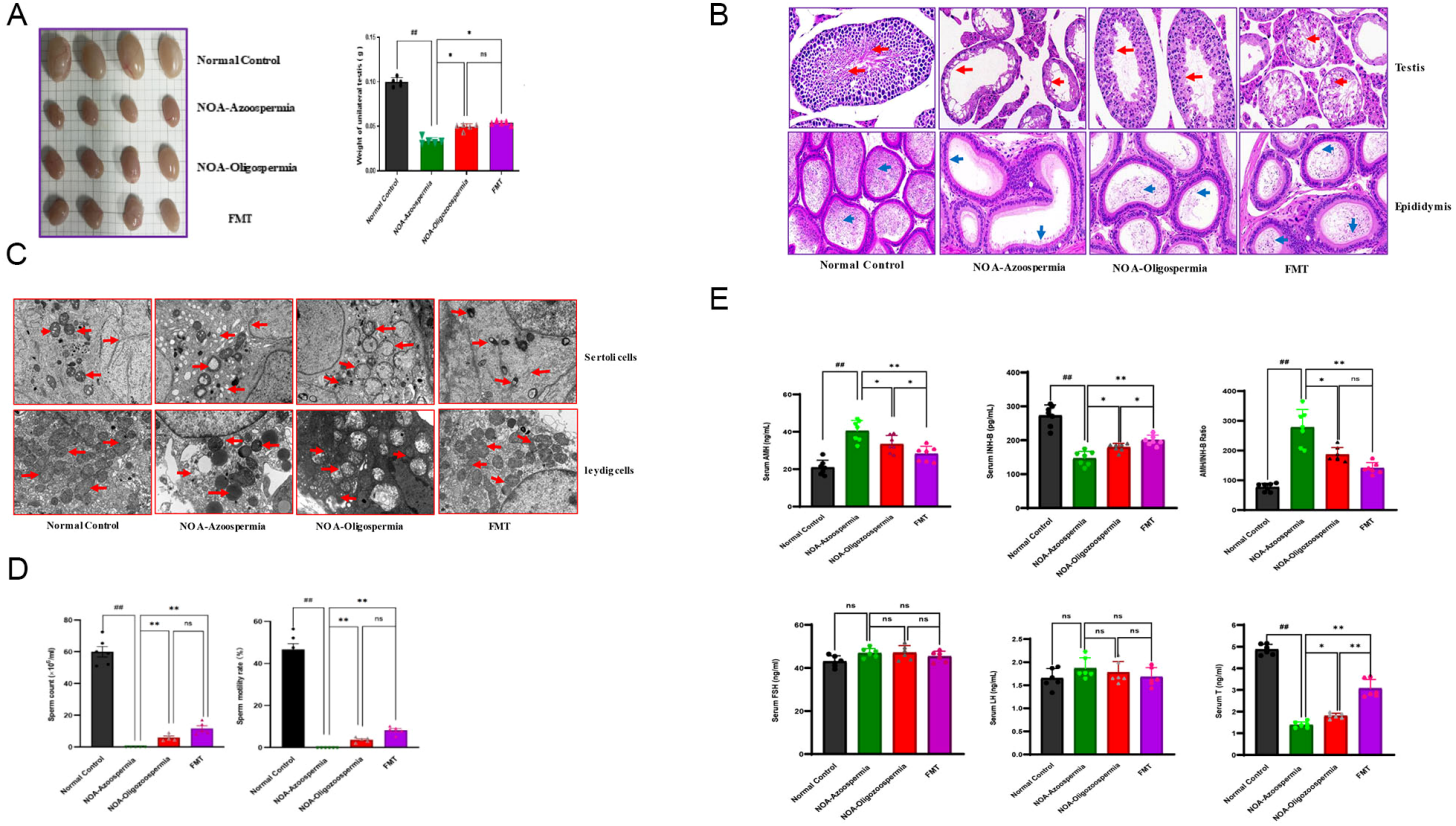
FMT rescues both systemic hormonal deficits and local testicular defects in the NOA mouse model. **(A)** Gross testicular morphology and quantification of testicular weight. **(B)** H&E staining of testicle. **(C)** TEM graph of testicle. **(D)** Sperm concentration and motility. **(E)** Serum hormone levels. Compared with the normal control group, Data are presented as mean ± SD, n=6-7. Statistical significance was determined by One-way ANOVA followed by Tukey’s *post-hoc* test. ^#^ *P* < 0.05, ^##^ *P* < 0.01 compared with the normal control group; * *P* < 0.05, ** *P* < 0.01 compared with the NOA-Azoospermia group.

Consequently, NOA mice exhibited a significant reduction in testicular weight compared with the control group, with a mean reduction of (31.81 ± 5.1)% (*P* < 0.01; **Figures 2A, B**, **Supplementary Figure 2**), indicating marked testicular atrophy induced by busulfan. Functionally, the epididymal sperm concentration in NOA mice was drastically reduced to (2.32 ± 1.58)×10^6^/mL, compared with (60.06 ± 5.96)×10^6^/mL in the control group, and sperm motility was nearly absent (mean motility=(3.73 ± 0.94)% vs (46.72± 5.29)% in the control group (*P* < 0.01; **Figure 2D**), demonstrating severe impairment of spermatogenic function.

Histologically, H&E staining revealed obvious seminiferous tubule atrophy, with a (58.7 ± 6.3)% reduction in the cross-sectional area of seminiferous tubules compared with controls, accompanied by severe loss of spermatogenic cells at all stages (the proportion of tubules with complete spermatogenic lineage was reduced from (91.2± 3.5)% in controls to (8.3 ± 2.1)% in NOA mice; **Figures 2B, C**). Electron microscopy further confirmed ultrastructural damage in Sertoli cells, including disrupted mitochondrial cristae and reduced tight junction integrity, as well as impairment of the blood-testis barrier (BTB), with a (41.5 ± 4.8)% decrease in the expression of BTB-related proteins (e.g., occludin) compared with the control group (**Figures 2B, C**).

Consistent with the phenotypic changes, serum hormone levels in the NOA model mice successfully mirrored clinical observations with quantifiable alterations: serum testosterone (T) levels were significantly decreased from 4.87 ± 0.21 ng/mL in controls to 1.38 ± 0.11 ng/mL (a 71.67% reduction, *P* < 0.01), and inhibin B (INH-B) levels were reduced from 272.91 ± 24.70 pg/mL to 52.15 ± 16.77 pg/mL (a 80.89% reduction, *P* < 0.01). Correspondingly, the AMH/INH-B ratio was increased by (65.18 ± 7.2)% (*P* < 0.01), while no significant changes were observed in follicle-stimulating hormone (FSH) (46.90 ± 1.67 ng/mL vs 43.01 ± 2.05 ng/mL in controls, *P*>0.05; **Figure 2E**) or luteinizing hormone (LH) levels (1.89 ± 0.19 ng/mL vs 1.65 ± 0.16 ng/mL in controls, *P*>0.05; **Figure 2E**). These quantifiable changes confirm the severe spermatogenic disorders and hormonal imbalances in the established NOA mouse model, laying a foundation for subsequent evaluation of FMT efficacy.

Subsequently, FMT was conducted to investigate the role of gut microbiota in spermatogenesis. The intervention resulted in a significant amelioration of spermatogenic dysfunction compared to untreated NOA mice, with quantifiable improvements in key indicators:unilateral testicular weight was restored by (37.03 ± 4.20)% (from 0.034 ± 0.002g in untreated NOA mice to 0.054 ± 0.001g in FMT-treated mice, *P* < 0.05; **Figure 2A**), epididymal sperm concentration increased from (2.32 ± 1.58)×10^6^/mL to (11.71 ± 3.15)×10^6^/mL (a 5.0-fold increase, *P* < 0.05), and sperm motility was elevated from (3.73 ± 0.94)% to (8.22 ± 1.29)% (*P* < 0.05; **Figures 2A, D**). Histologically, spermatogenic cells of various stages reappeared within the seminiferous tubules, with the proportion of tubules containing complete spermatogenic lineage increasing from (8.3 ± 2.1)% (untreated NOA) to (45.6 ± 5.3)% (FMT-treated), and the ultrastructure of Sertoli cells was improved, with a (35.2 ± 4.6)% recovery in the expression of BTB-related proteins (**Figures 2B, C**).Meanwhile, serum levels of hormones were also significantly restored: serum T levels increased from 1.59 ± 0.21 ng/mL (untreated NOA) to 3.07 ± 0.36 ng/mL (a 48.21% recovery rate), and INH-B levels rose from 52.15 ± 16.77 pg/mL to 193.22 ± 12.38 pg/mL (a 73.01% recovery rate, *P* < 0.05; **Figure 2E**). These results demonstrate that modulation of the gut microbiota could reverse both systemic hormonal deficits and local testicular defects of spermatogenic dysfunction in NOA mice, with quantifiable effect sizes confirming the efficacy of FMT intervention. Collectively, these findings validate the animal model as a clinically relevant platform for subsequent mechanistic and interventional studies.

### 3.3 Analysis of the mechanism of the “intestine-spermatic cord axis” in regulating spermogenesis

#### 3.3.1 Gut microbiota dysbiosis in NOA mice

**Figure 3:**
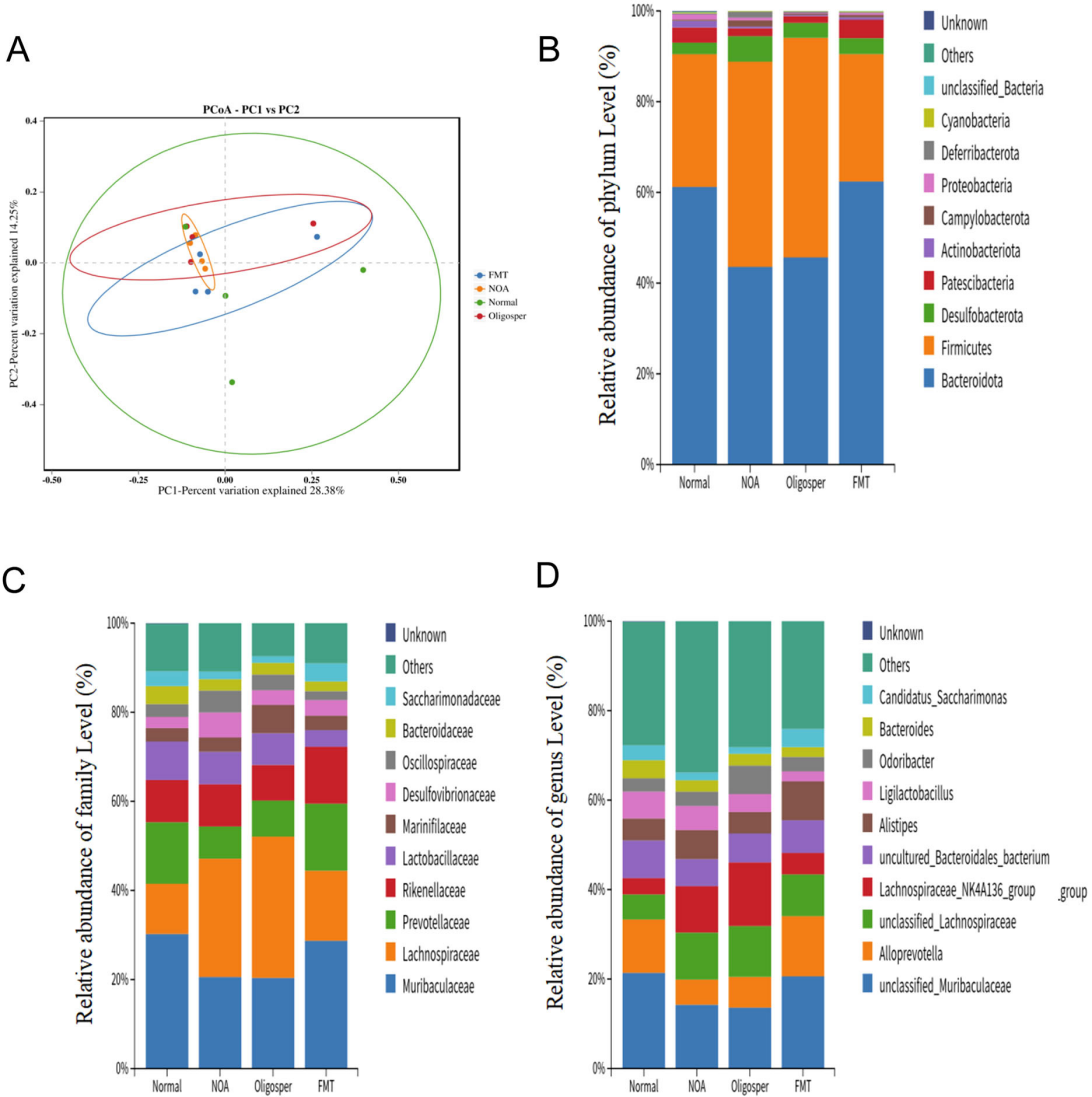
Gut microbiota dysbiosis in NOA mice and its reversal by FMT. **(A)** PCoA analysis chart; **(B-D)** Relative abundance of phylum level **(B)**, family level **(C)** and genus level **(D)**.

To investigate the role of gut microbiota in the NOA model, 16S rRNA sequencing was performed. PCoA analysis revealed a significant structural shift of the gut microbiota in the NOA mice compared to the normal control, while FMT intervention restored it to a composition close to that of the normal group (**Figure 3A**). At the phylum level, the most notable alteration was a significant increase in the *Firmicutes*/*Bacteroidetes* (F/B) ratio, a hallmark of microbial dysbiosis, in the NOA model group, which was effectively reversed by FMT (**Figure 3B**). Further analysis indicated that this dysbiosis was primarily driven by changes in two key taxonomic units. At the family level, the relative abundance of *Muribaculaceae* was drastically reduced in the NOA model (**Figure 3C**). Similarly, at the genus level, the abundance of *Bacteroides* was significantly decreased (**Figure 3D**). Importantly, FMT intervention markedly restored the abundance of these two critical bacterial groups (**Figures 3C, D**). Further taxonomic analysis at the class, order, and species levels, along with a Venn analysis of shared and unique features, are provided in **Supplementary Figure 3**.

These results demonstrate that NOA is associated with a distinct gut microbiota dysbiosis, characterized by an increased F/B ratio and a specific depletion of *Muribaculaceae* and *Bacteroides*, which can be effectively reversed by FMT.

#### 3.3.2 Correlation between key gut microbiota and reproductive function parameters

**Figure 4:**
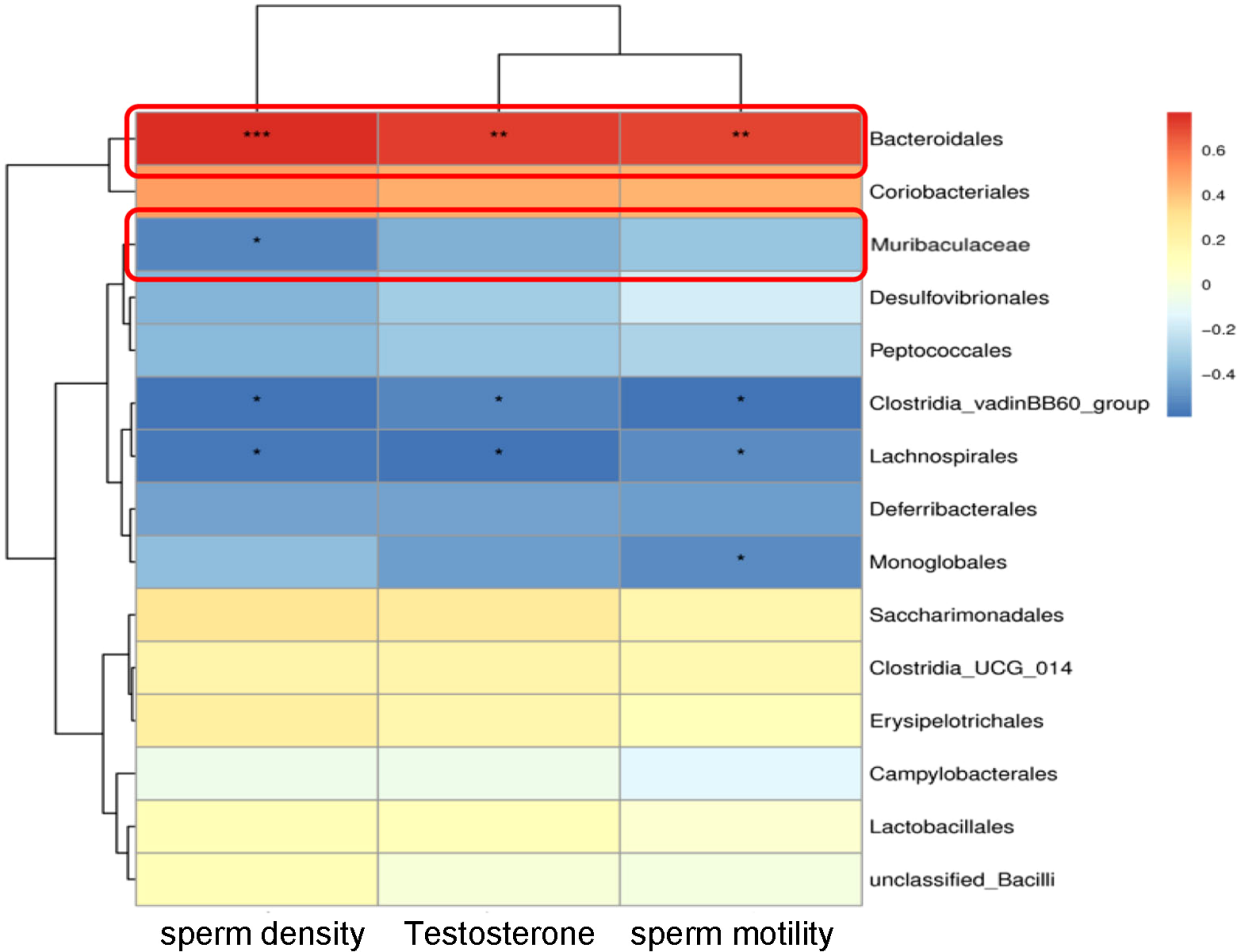
Correlation heatmap between gut microbiota and spermatogenic parameters. Red indicates a positive correlation, and blue indicates a negative correlation. The color intensity corresponds to the correlation coefficient. * *P* < 0.05, ** *P* < 0.01, *** *P* < 0.001.

We next analyzed the correlation between testicular functional parameters and the key altered gut microbiota in NOA mice. Specifically, *Bacteroidales* at the order level exhibited strong positive correlations with sperm density, T level, and sperm motility (*P* < 0.001, **Figure 4**). Concurrently, the abundance of *Muribaculaceae* at the family level was also significantly and positively correlated with sperm density (*P* < 0.05, **Figure 4**). Critically, to directly validate the core indicator of our clinical predictive model, we performed a correlation analysis between the serum AMH/INH-B ratio and the abundance of these key bacterial taxa. Some other taxa, such as *Desulfovibrionales* and *Peptococcales*, showed a negative correlation trend, although not statistically significant.These findings establish a statistically significant positive association between the abundance of beneficial bacterial taxa (*Bacteroidales* and *Muribaculaceae*) and key metrics of reproductive function, underscoring their potential role in maintaining testicular health.

#### 3.3.3 Impact of gut microbiota on the local testicular microenvironment

**Figure 5:**
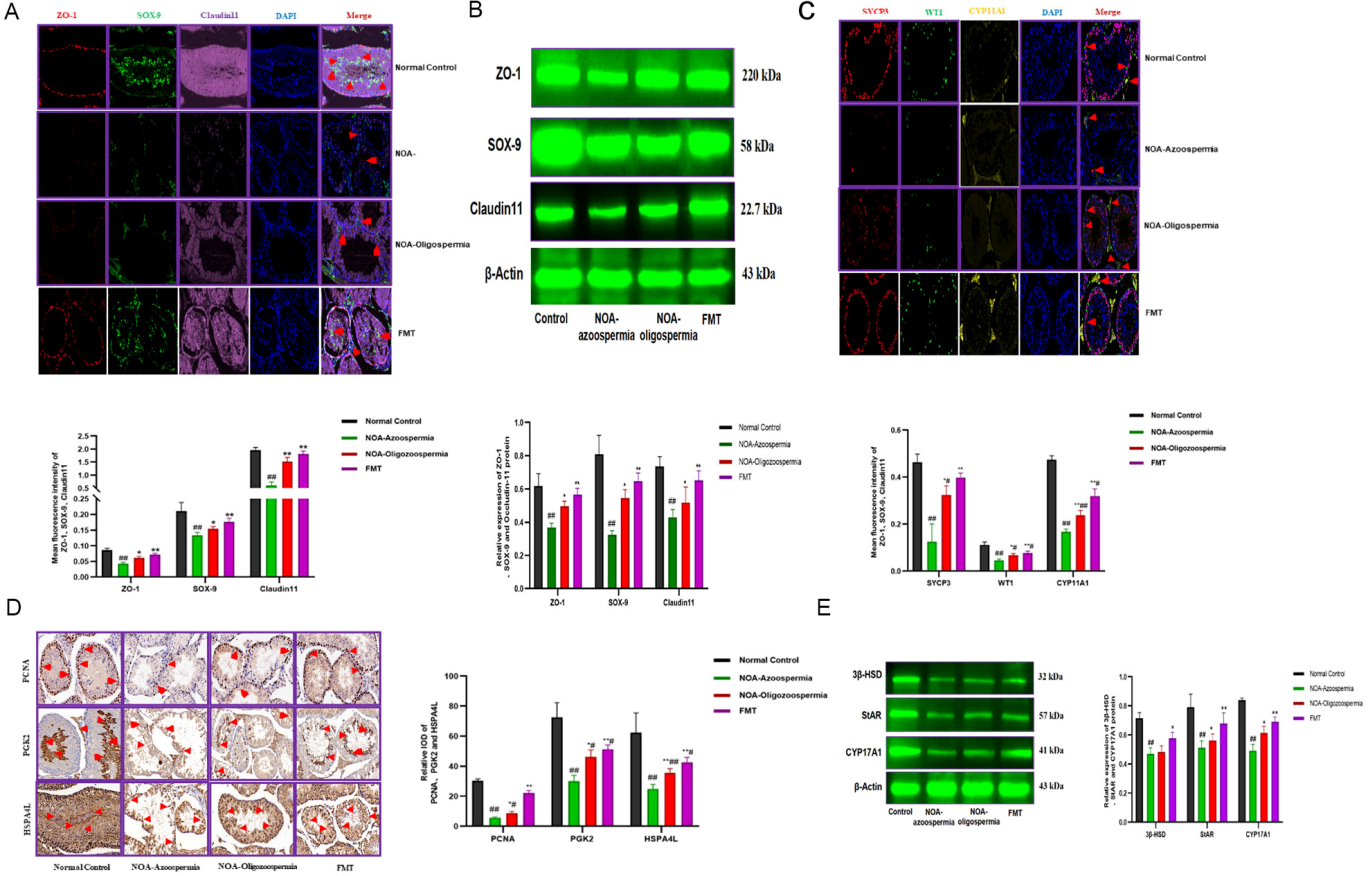
FMT rescues key molecular mechanisms of spermatogenesis. **(A, B)** Immunofluorescence **(A)** and Western Blot **(B)** of tight junction proteins ZO-1, SOX-9 and Claudin11 in the BTB of mice with normal FSH levels across various groups. **(C)** Immunofluorescence of SYCP3, WT1 and CYP11A1 in the testes of mice with normal FSH levels in each group. **(D)** Immunohistochemical of PCNA, PGK2 and HSPA4L proteins in the seminiferous tubules and epididymis of FSH-normal mice. **(E)** Western Blot of key testosterone secreting enzymes 3β-HSD, StAR and CYP17A1 in Leydig cells of testicular tissue of FSH normal mice. For all quantification plots, data are presented as mean ± SEM, n=5. Statistical significance was determined by One-way ANOVA. ^#^ *P* < 0.05, ^##^ *P<*0.01 compared with the normal control group; * *P* < 0.05, ** *P* < 0.01 compared with the NOA-Azoospermia group.

To investigate the impact of gut microbiota on the local testicular microenvironment, we assessed the integrity of the BTB and key processes in spermatogenesis. The results showed that the BTB proteins ZO-1, Claudin11, and their key transcriptional regulator SOX-9 were significantly downregulated in NOA model mice (*P* < 0.05;n=5 **Figures 5A, B**). Importantly, FMT intervention markedly upregulated the expression of these proteins and partially restored their continuous localization at the basal compartment of the seminiferous tubules (*P* < 0.01; **Figures 5A, B**). Analysis of stage-specific markers revealed that the expression of PCNA (a marker of spermatogonia proliferation) and SYCP3/PGK2 (markers for meiosis in spermatocytes) (Zheng et al., 2024; Miura et al., 2002; Park et al., 2018) were severely suppressed in the NOA model (*P* < 0.05; **Figures 5C, D**), and were significantly restored by FMT (*P* < 0.05; **Figures 5C, D**). Furthermore, Western blot analysis revealed that the protein levels of the steroidogenic enzymes StAR and CYP17A1 in Leydig cells were significantly reduced in the NOA model (*P* < 0.05; **Figure 5E**) and markedly upregulated following FMT intervention (*P* < 0.05; **Figure 5E**). These results suggest that the restoration of gut microbiota is closely linked to the recovery of the BTB integrity and steroidogenic function.

### 3.4 Gut microbiota-derived SCFAs are associated with testicular function recovery

**Figure 6:**
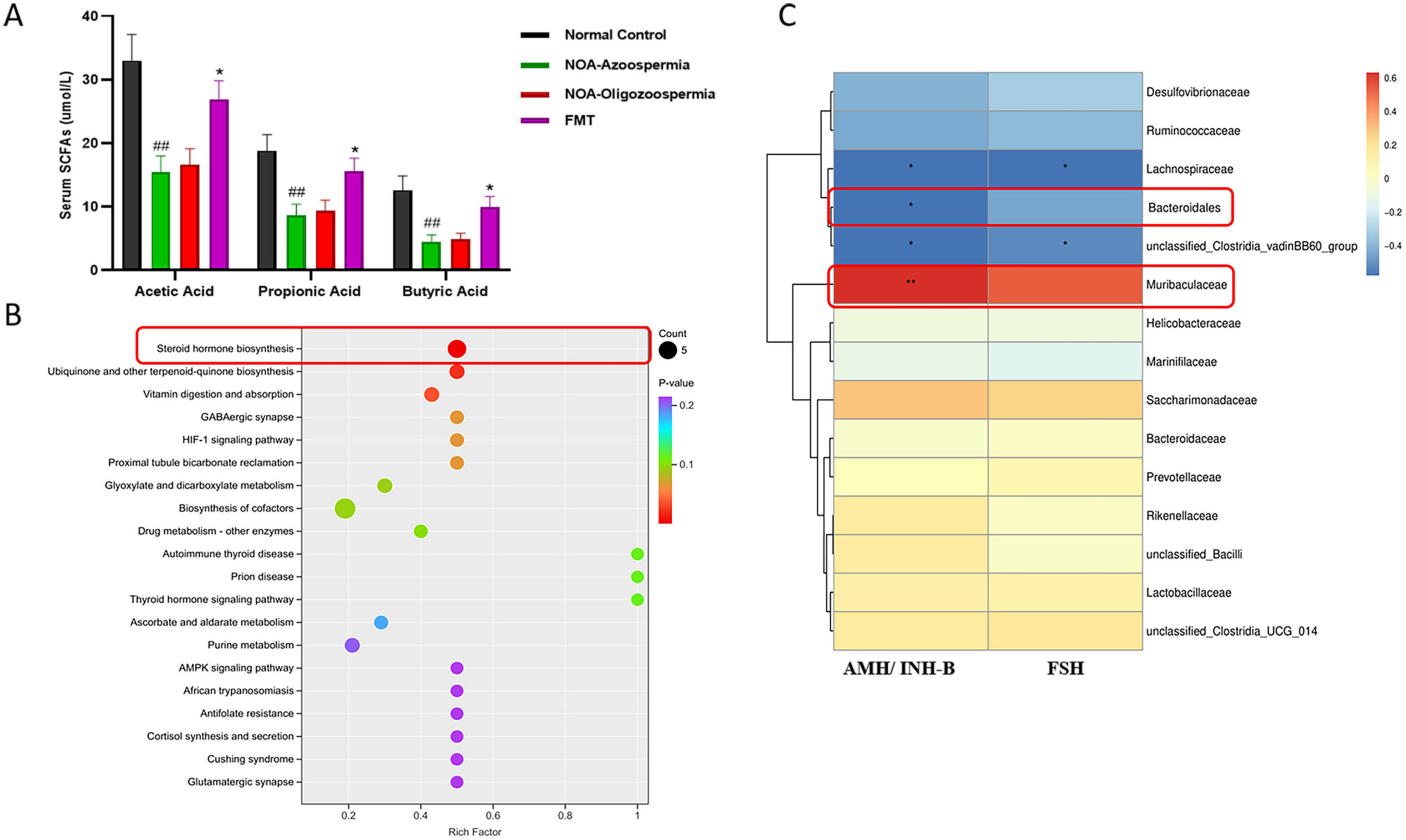
Gut microbiota-derived metabolites and their association with testicular function and clinical markers. **(A)** Serum short-chain fatty acid (SCFA) levels across experimental groups. **(B)** KEGG pathway enrichment analysis of differentially expressed metabolites. **(C)** Spearman correlation heatmap between the serum AMH/INH-B ratio, FSH, and key differential gut microbiota. Data are presented as mean ± SEM. Statistical significance was determined by One-way ANOVA for SCFA levels, Fisher’s exact test for pathway enrichment analysis, and Spearman’s rank correlation test for correlation analysis. ^##^ *P* < 0.01 compared with the normal control group; * *P* < 0.05 compared with the NOA group.

To further elucidate the mechanism by which gut microbiota influences spermatogenesis, we analyzed serum SCFA levels and performed metabolomic pathway enrichment analysis. Metabolomic analysis revealed that the concentrations of acetic acid, propionic acid, and butyric acid were markedly reduced in the NOA model group compared to the control group (*P* < 0.01; **Figure 6A**). Following FMT treatment, all three SCFAs were significantly restored to levels comparable to those in the NOA group (*P* < 0.05; **Figure 6A**). KEGG pathway enrichment analysis of differentially expressed metabolites revealed significant alterations in the “Steroid hormone biosynthesis” pathway (*P* < 0.01; **Figure 6B**), suggesting that gut microbiota-derived SCFAs may be metabolically linked to testicular steroidogenesis and could partially underpin the recovery of testosterone synthesis following FMT. Furthermore, correlation analysis demonstrated a strong positive association between the serum AMH/INH-B ratio and the relative abundance of gut *Muribaculaceae* (*P* < 0.01; **Figure 6C**), supporting a potential correlative mechanistic connection between key reproductive serological markers and core microbial signatures in this model.

### 3.5 ROC curves of serum AMH/INH-B ratio and *Bacteroidales* abundance used alone or in combination for predicting sperm retrieval outcomes in FSH-normal NOA mice

**Figure 7:**
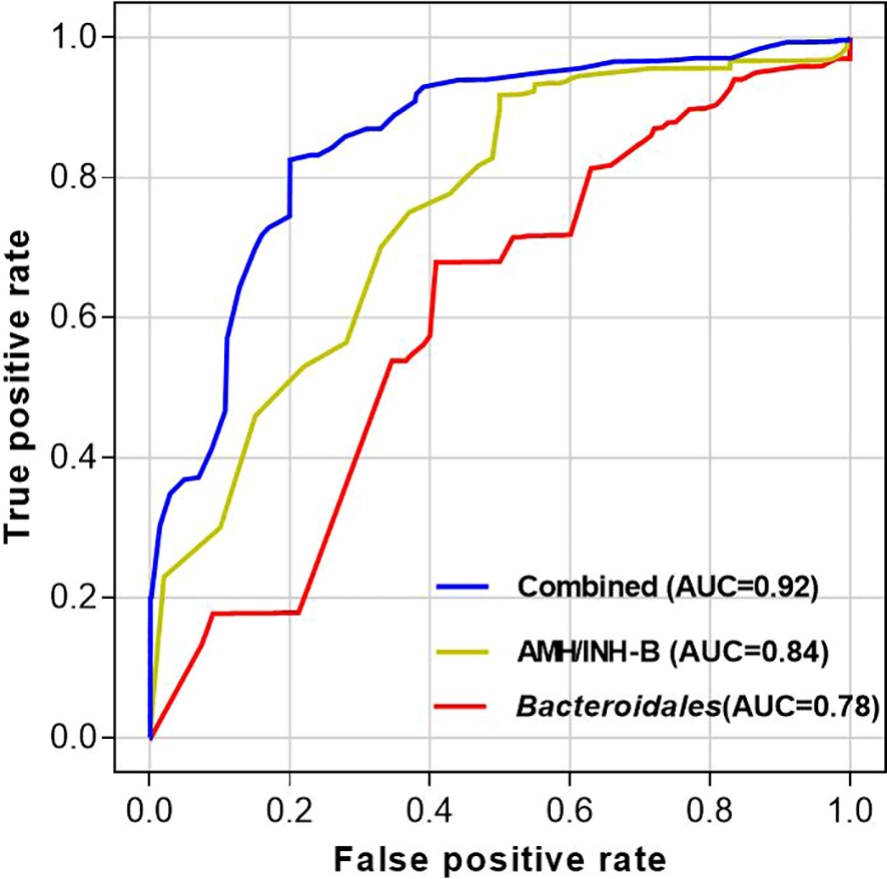
ROC curves of serum AMH/INH-B ratio, *Bacteroidales* abundance, and their combined application.

The areas under the ROC curves (AUC) for predicting sperm retrieval outcomes using the Serum AMH/INH-B Ratio, *Bacteroidales* Abundance, and the combination of both indicators were 0.84, 0.78, and 0.92, respectively. The sensitivities were 82.5%, 75.0%, and 88.2%, and the specificities were 78.9%, 72.4%, and 85.7%, respectively. Combining the two indicators can enhance the predictive efficacy for sperm retrieval, providing important reference for clinical optimization of surgical indications and prediction of surgical outcomes (*P* < 0.05; **Table 2**, **Figure 7**).

**Table 2:** Performance metrics of different models for discriminating NOA-Azoospermia from NOA-Oligospermia in FSH-normal NOA mice.

Model	AUC (95% CI)	Cut-off value	Sensitivity (%)	Specificity (%)	Youden’s index
AMH/INH-B Ratio	0.84 (0.75-0.93)	0.52	82.5	78.9	0.614
*Bacteroidales* Abundance	0.78 (0.66-0.90)	0.34	75.0	72.4	0.474
Combined Model	0.92 (0.86-0.98)	0.65 *	88.2	85.7	0.739

*Predicted probability derived from multivariate logistic regression incorporating both AMH/INH-B ratio and *Bacteroidales* abundance.

AUC, Area Under the Curve; CI, Confidence Interval.

## 4 Discussion

### 4.1 Key findings and novelty

This study establishes a novel, non-invasive predictive model for sperm retrieval success in FSH-normal NOA patients by integrating the serum AMH/INH-B ratio with gut *Bacteroidales* and *Muribaculaceae* abundance. This multi-dimensional approach significantly improves pre-operative decision-making, potentially reducing unnecessary micro-TESE procedures and their associated burdens. While testicular biopsy remains the diagnostic standard, its variable success rates (40-60%) in this population highlight the need for better predictors. Our model addresses this gap by leveraging complementary biomarkers, offering comparable accuracy without surgical risks. The clinical relevance of these findings is strengthened by successful validation in a busulfan-induced FSH-normal NOA mouse model that mimics the human endocrine profile.

### 4.2 Mechanistic insights into the gut-testis axis

Our findings illuminate a potential causal mechanism link along the gut-SCFA-testis axis. The specific depletion of *Bacteroidales* and *Muribaculaceae* in NOA was associated with significantly reduced serum SCFAs (acetate, propionate, and butyrate) (Guo et al., 2026). These microbial metabolites appear to function as critical signaling molecules connecting gut ecology to testicular function, as evidenced by the parallel restoration of SCFA levels and BTB proteins (ZO-1, Claudin11) following FMT. Furthermore, KEGG pathway analysis identified ‘Steroid hormone biosynthesis’ as a key microbiome-affected pathway, which aligns with the observed upregulation of testosterone synthesis enzymes (StAR, CYP17A1) after FMT. This integrated evidence supports a model where gut microbiota derived SCFAs coordinate both the structural integrity of the testicular microenvironment and its endocrine function, though direct molecular mechanisms remain to be elucidated (Mann et al., 2024; Hu et al., 2023).

Building upon this model, our study delineates a multi-pathway synergistic mechanism by which SCFAs regulate male reproductive function. We propose that SCFAs exert multi-target regulation through direct actions on testicular cells and indirect modulation of systemic metabolism and immune status. At the cellular level, SCFAs directly upregulate the expression and membrane localization of tight junction proteins (ZO-1, Claudin11) in Sertoli cells, thereby repairing BTB integrity, which aligns with the emerging concept of gut-derived metabolites regulating reproductive barriers. Concurrently, SCFAs promote the transcription and translation of CYP17A1 in Leydig cells, enhancing testosterone synthesis capacity.This integrated action underscores the central role of SCFAs in coordinating testicular microenvironment homeostasis (Hays et al., 2024).

### 4.3 Clinical implications

Our study delineates a pathophysiological feature of FSH-normal NOA centered on Sertoli cell dysfunction, interconnected with gut microbiota dysbiosis. While FSH levels are normal, reduced INH-B and AMH indicate Sertoli cell impairment (Shan et al., 2025; Zhou et al., 2025), with the AMH/INH-B ratio serving as a superior marker of the testicular microenvironment (Kato et al., 2022; Holt et al., 2023). This testicular pathology is linked to the depletion of gut *Bacteroidales* and *Muribaculaceae*, though the exact downstream pathways connecting gut dysbiosis to testicular dysfunction require further targeted experimentation.This study used ROC curve analysis to show that the AUC values for Serum AMH/INH-B Ratio, *Bacteroidales* Abundance, and the combined detection of these two indicators were 0.84, 0.78, and 0.92, respectively. Relevant studies have found that FSH, AMH, and INH-B are all associated with spermatogenic function, but there are individual differences. The diagnostic efficacy of single detection of the Serum AMH/INH-B Ratio and intestinal *Bacteroidales* Abundance for non-obstructive azoospermia (NOA) is unsatisfactory. This study demonstrated that when the two indicators were combined for the diagnosis of NOA, the AUC value was 0.92, with a sensitivity of 88.2% and a specificity of 85.7%. This indicates that the combined detection of the serum AMH/INH-B ratio and *Bacteroides* abundance is of high value in the classification of non-obstructive azoospermia in patients with FSH-normal levels.

Critically, our study provides compelling functional evidence for the causal role of gut microbiota, as FMT successfully reversed spermatogenic dysfunction in the NOA mouse model. This comprehensive recovery across testicular histology, BTB integrity, and systemic hormone levels demonstrates that the pathology represents a reversible, microbiota-dependent dysregulation. The therapeutic mechanism involves multiple coordinated pathways. First, the restoration of SCFAs (acetate, propionate, and butyrate) exerts potent anti-inflammatory effects by inhibiting the NF-κB signaling pathway and promoting regulatory T cell expansion, thereby reestablishing immune homeostasis (Kaltsas et al., 2025; Zhang et al., 2024; Yu et al., 2025; Wu et al., 2024). Second, FMT modulates neuroendocrine signaling along the HPG axis by regulating neurotransmitters including GABA and serotonin, which helps restore the balance between inhibitory and excitatory signals, optimizes pulsatile GnRH release, and enhances gonadotropin production and spermatogenic efficiency (Organski et al., 2025). Third, FMT strengthens gut barrier integrity through enhanced expression of tight junction proteins, reducing endotoxin translocation and systemic inflammation, thus alleviating indirect damage to the BTB. Notably, while current applications of FMT in reproductive medicine are predominantly focused on modulating female fertility or managing immunotherapy-related colitis in cancer patients, our findings are among the first to demonstrate its therapeutic efficacy in a male infertility context, specifically for NOA with normal FSH, highlighting its innovative potential as a non-hormonal therapeutic avenue.

In terms of clinical translation, the serum AMH/INH-B ratio holds significant promise as a functional biomarker for Sertoli cell function. Our novel finding of its correlation with gut-derived SCFAs extends its application beyond static diagnosis, positioning it as a potential non-invasive surrogate for monitoring gut-testis axis activity and assessing the efficacy of microbiota-targeted interventions (Kong et al., 2021). This proposition is strongly supported by the growing consensus, as outlined in recent authoritative reviews, that the gut microbiota regulates male reproductive disorders through sex hormone pathways, thereby providing a causal foundation for therapeutic strategies targeting the gut-testis axis (Lv et al., 2024; Wang et al., 2024; Liu et al., 2024). Therefore, by linking a specific hormonal signature with gut microbial metabolic output, our work translates the conceptual gut-testis axis into a practical framework for therapeutic monitoring, advancing the path toward personalized management of male infertility.

### 4.4 Limitations

This study offers several key innovations, including its focus on the understudied FSH-normal NOA cohort, the development of a multi-dimensional predictive model, and the functional validation of the gut-testis axis via FMT. However, this study has several limitations that should be acknowledged.First, regarding the study design, the relatively small sample size from a single center (58 patients, with an imbalanced subgroup size of SRP = 24 and SRN = 10) may limit the statistical power and introduce potential selection bias.The retrospective, single-center design also substantially limits the generalizability of our findings. Future large-scale, multicenter prospective studies are essential to validate and extend the clinical applicability of our proposed non-invasive predictive model. A proposed framework for future multicenter validation includes a prospective study design with standardized protocols for patient recruitment, sample collection, and microbiome analysis across multiple centers. Second, in terms of research scope, this study focused on the relationship between serum INH-B, AMH, the AMH/INH-B ratio, and gut *Bacteroides* abundance(in mice). Other potential biomarkers, such as seminal plasma microRNAs and long non-coding RNAs, were not investigated and may offer additional predictive value. Incorporating these multi-omics biomarkers in future research could further refine prediction accuracy. Third, gut microbiota analysis was only performed in the mouse model, not in the clinical patient cohort. Directly extrapolating the mouse *Bacteroidales*/*Muribaculaceae* findings to support a clinical predictive model involving gut *Bacteroidales* abundance represents an unjustified cross-species inference without direct clinical validation; thus, the role of Bacteroidales in human NOA requires future clinical investigation.Fourth, concerning mechanistic exploration, our study demonstrates a correlation between gut microbiota restoration and testicular improvement but does not fully establish direct causality. Although we observed that FMT restored SCFA levels and correlated these changes with testicular protein expression, the specific signaling pathways through which SCFAs exert their effects remain unclear.Additionally, the therapeutic efficacy of FMT may be influenced by individual variability in host microbiota composition, immune status, and genetic background, underscoring the need for more detailed preclinical studies and rigorously designed clinical trials to translate these findings into safe and effective microbiome-based therapies. Finally, the retrospective nature of the human study is a key limitation, which we explicitly acknowledge here.

## 5 Conclusions

**Figure 8:**
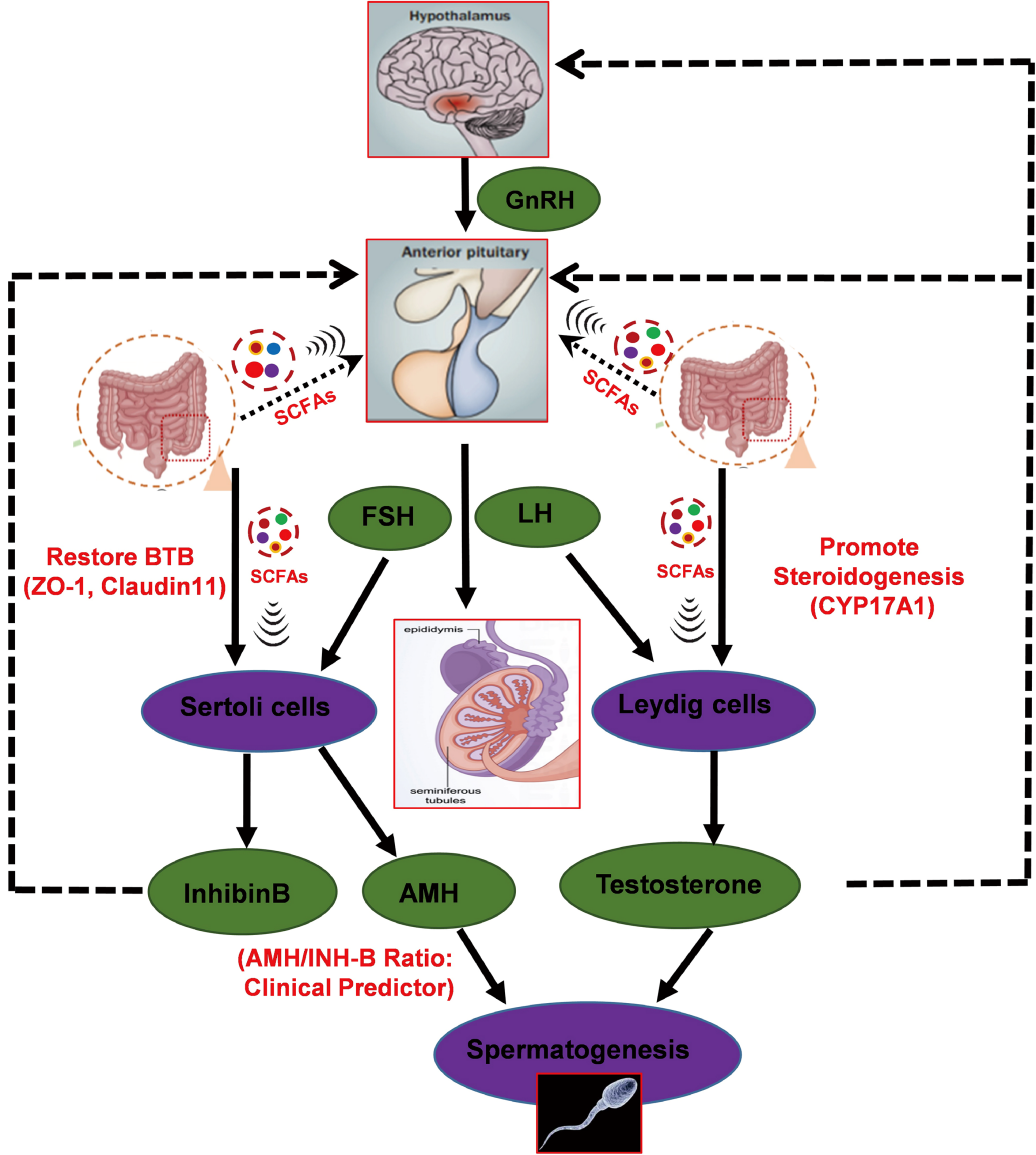
Proposed working model of the gut microbiota-brain-testicular (GBT) axis in regulating spermatogenesis. This schematic illustrates the proposed mechanism wherein gut microbiota dysbiosis acts as an upstream driver of spermatogenic failure in FSH-normal NOA. Dysbiosis-induced alterations in microbial metabolites are hypothesized to disrupt the hypothalamic-pituitary-gonadal (HPG) axis, leading to impaired Sertoli and Leydig cell function, and ultimately, spermatogenic failure.

In summary, this study establishes a novel, non-invasive predictive model for sperm retrieval success in FSH-normal NOA patients by integrating the serum AMH/INH-B ratio with the abundance of gut *Bacteroides* and *Muribaculaceae*. This combined approach holds significant promise for improving personalized clinical decision-making and avoiding unnecessary surgical interventions. The functional recovery observed in an animal model following FMT substantiates a critical role for the gut-testis axis in male infertility. Based on these findings, a working model is proposed where gut microbiota dysbiosis functions as an upstream driver of spermatogenic failure, achieved by impairing both the local testicular microenvironment and systemic endocrine function (**Figure 8**). Future large-scale, multicenter prospective studies are warranted to validate this predictive model. Furthermore, pursuing mechanistic insights through multi-omics approaches will be essential for the development of targeted microbiome-based therapies, such as next-generation probiotics, which represent a promising, non-hormonal therapeutic avenue for male infertility.

## Data Availability

The original contributions presented in the study are included in the article/**Supplementary Material**. Further inquiries can be directed to the corresponding author.

## Supplementary material

Supplementary Figure 1 Normal Karyotype of an FSH-normal NOA patient.

Supplementary Figure 2 Sperm morphology in different experimental groups.

Supplementary Figure 3 Detailed analysis of gut microbiota dysbiosis and its reversal by FMT. **(A)** Veen analysis chart; **(B-D)** Relative abundance of class level **(B)**, order level **(C)** and species level **(D)**.

## References

[B8] Bazzar B. Reshadfar E. Namdar P. Pourbagher D. Lafmejani R. B. Soleimanzadeh A. (2026). The relationship between seminal microbiome and male reproductive wellness: a systematic review. Biol. Reprod. 114, 1151–1167. doi: 10.1093/biolre/ioag003. PMID: 41615012

[B7] Borzan V. Riedl R. Obermayer-Pietsch B. (2023). Probiotic vs. placebo and metformin: probiotic dietary intervention in polycystic ovary syndrome-a randomized controlled trial. BMC Endocr. Disord. 23, 82. doi: 10.1186/s12902-023-01294-6. PMID: 37062834 PMC10106320

[B12] Cannarella R. Crafa A. Curto R. Condorelli R. A. La Vignera S. Calogero A. E. (2024). Obesity and male fertility disorders. Mol. Asp. Med. 97, 101273. doi: 10.1016/j.mam.2024.101273. PMID: 38593513

[B10] Cavallini G. Beretta G. Biagiotti G. (2021). Preliminary study of letrozole use for improving spermatogenesis in non-obstructive azoospermia patients with normal serum FSH. Asian J. Androl. 13, 895–897. doi: 10.1038/aja.2011.44. PMID: 21706040 PMC3739567

[B6] Driuchina A. Hintikka J. Lehtonen M. Keski-Rahkonen P. O'Connell T. Juvonen R. . (2023). Identification of gut microbial lysine and histidine degradation and CYP-dependent metabolites as biomarkers of fatty liver disease. mBio 14, e0266322. doi: 10.1128/mbio.02663-22. PMID: 36715540 PMC9973343

[B20] Guo L. Pei X. Tan J. Sun H. Jiang S. Wei H. . (2026). From association to intervention: Muribaculaceae driven SCFAs production enhances boar semen quality via inflammation alleviation. NPJ Biofilms Microbiomes. 12, 69. doi: 10.1038/s41522-026-00933-9. PMID: 41698951 PMC13021910

[B23] Hays K. E. Pfaffinger J. M. Ryznar R. (2024). The interplay between gut microbiota, short-chain fatty acids, and implications for host health and disease. Gut. Microbes 16, 2393270. doi: 10.1080/19490976.2024.2393270. PMID: 39284033 PMC11407412

[B27] Holt R. Yahyavi S. K. Kooij I. Andreassen C. H. Andersson A. M. Juul A. . (2023). Low serum anti-Müllerian hormone is associated with semen quality in infertile men and not influenced by vitamin D supplementation. BMC Med. 21, 79. doi: 10.1186/s12916-023-02782-1. PMID: 36855109 PMC9976369

[B22] Hu C. Xu B. Wang X. Wan W. Lu J. Kong D. . (2023). Gut microbiota-derived short-chain fatty acids regulate group 3 innate lymphoid cells in HCC. Hepatology 77, 48–64. doi: 10.1002/hep.32449. PMID: 35262957 PMC9970019

[B1] Hubbard L. Rambhatla A. Glina S. (2025). Nonobstructive azoospermia: an etiologic review. Asian J. Androl. 27, 279–287. doi: 10.4103/aja202472. PMID: 39243180 PMC12112933

[B9] Kaltsas A. Giannakodimos I. Markou E. Stavropoulos M. Deligiannis D. Kratiras Z. . (2025). The androbactome and the gut microbiota-testis axis: a narrative review of emerging insights into male fertility. Int. J. Mol. Sci. 26 (13), 6211. doi: 10.3390/ijms26136211. PMID: 40649988 PMC12249747

[B11] Kaltsas A. Stavros S. Kratiras Z. Zikopoulos A. Machairiotis N. Potiris A. . (2024). Predictors of successful testicular sperm extraction: a new era for men with non-obstructive azoospermia. Biomedicines 12 (12), 2679. doi: 10.3390/biomedicines12122679. PMID: 39767586 PMC11726830

[B26] Kato T. Mizuno K. Matsumoto D. Nishio H. Nakane A. Kurokawa S. . (2022). Low serum inhibin B/follicle-stimulating hormones and anti-Müllerian hormone/follicle-stimulating hormones ratios as markers of decreased germ cells in infants with bilateral cryptorchidism. J. Urol. 207 (3), 701–709. doi: 10.1097/JU.0000000000002344. PMID: 34823367 PMC12721657

[B3] Klami R. Tomás C. Mankonen H. Perheentupa A. (2024). ICSI outcome after microdissection testicular sperm extraction, testicular sperm aspiration and ejaculated sperm. Reprod. Biol. 24, 100825. doi: 10.1016/j.repbio.2023.100825. PMID: 38000348

[B13] Kong X. Ye Z. Chen Y. Zhao H. Tu J. Meng T. . (2021). Clinical application value of inhibin B alone or in combination with other hormone indicators in subfertile men with different spermatogenesis status: a study of 324 Chinese men. J. Clin. Lab. Anal. 35, e23882. doi: 10.1002/jcla.23882. PMID: 34181290 PMC8373365

[B34] Liu J. L. Chen L. J. Liu Y. Li J. H. Zhang K. K. Hsu C. . (2024). The gut microbiota contributes to methamphetamine-induced reproductive toxicity in male mice. Ecotoxicol. Environ. Saf. 279, 116457. doi: 10.1016/j.ecoenv.2024.116457. PMID: 38754198

[B32] Lv S. Huang J. Luo Y. Wen Y. Chen B. Qiu H. . (2024). Gut microbiota is involved in male reproductive function: a review. Front. Microbiol. 15, 1371667. doi: 10.3389/fmicb.2024.1371667. PMID: 38765683 PMC11099273

[B21] Mann E. R. Lam Y. K. Uhlig H. H. (2024). Short-chain fatty acids: linking diet, the microbiome and immunity. Nat. Rev. Immunol. 24, 577–595. doi: 10.1038/s41577-024-01014-8. PMID: 38565643

[B18] Miura C. Miura T. Yamashita M. (2002). PCNA protein expression during spermatogenesis of the Japanese eel (Anguilla japonica). Zool. Sci. 19, 87–91. doi: 10.2108/zsj.19.87. PMID: 12025409

[B31] Organski A. C. Rajwa B. Reddivari A. Jorgensen J. S. Cross T. L. (2025). Gut microbiome-driven regulation of sex hormone homeostasis: a potential neuroendocrine connection. Gut. Microbes 17, 2476562. doi: 10.1080/19490976.2025.2476562. PMID: 40071861 PMC11913384

[B5] Ozer C. Hasirci E. Ceyhan E. Kayra M. V. Sarıturk C. Goren M. R. (2024). Microdissection testicular sperm extraction in non-obstructive azoospermic patients with solitary testis: a retrospective case-control study. Rev. Int. Androl. 22, 17–22. doi: 10.22514/j.androl.2024.003. PMID: 38735873

[B19] Park H. J. Lee W. Y. Park C. Hong K. H. Kim J. H. Song H. (2018). Species-specific expression of phosphoglycerate kinase 2 (PGK2) in the developing porcine testis. Theriogenology 110, 158–167. doi: 10.1016/j.theriogenology.2018.01.007. PMID: 29407897

[B16] Qi L. Liu Y. P. Zhang N. N. Su Y. C. (2021). Predictors of testicular sperm retrieval in patients with non-obstructive azoospermia: a review. J. Int. Med. Res. 49, 1–14. doi: 10.1177/03000605211002703. PMID: 33794677 PMC8020245

[B14] Ran L. Gao Z. Chen Q. Cui F. Liu X. Xue B. (2023). Identification and validation of diagnostic signature genes in non-obstructive azoospermia by machine learning. Aging 15, 4465–4480. doi: 10.18632/aging.204749. PMID: 37227814 PMC10257997

[B15] Schwarzkopf V. Wistuba J. Sandhowe-Klaverkamp R. Kliesch S. Gromoll J. Schubert M. (2025). Unraveling a subgroup of men with unexplained male infertility-men with normogonadotropic nonobstructive azoospermia. J. Clin. Endocrinol. Metab. 110, 3400–3411. doi: 10.1210/clinem/dgaf200. PMID: 40231331 PMC12623054

[B4] Shah R. Agarwal A. Kavoussi P. Rambhatla A. Saleh R. Cannarella R. . (2023). Consensus and diversity in the management of varicocele for male infertility: results of a global practice survey and comparison with guidelines and recommendations. World J. Mens. Health 41, 164–197. doi: 10.5534/wjmh.220048. PMID: 35791302 PMC9826919

[B24] Shan L. Guo X. Hu Y. Zhou H. Meng X. Liu K. . (2025). L-citrulline protects testicular Sertoli cell function by mitigating DNA damage via the gut-testis axis of sheep fed a high-concentrate diet. NPJ Biofilms Microbiomes 11, 202. doi: 10.1038/s41522-025-00832-5. PMID: 41168195 PMC12575620

[B33] Wang L. Han Q. Liu Y. Ma X. Han H. Yan L. . (2024). Activation of aryl hydrocarbon receptor protein promotes testosterone synthesis to alleviate abnormal spermatogenesis caused by cholestasis. Int. J. Biol. Macromol. 282, 136478. doi: 10.1016/j.ijbiomac.2024.136478. PMID: 39393744

[B2] Wang W. Su L. Meng L. He J. Tan C. Yi D. . (2023). Biallelic variants in KCTD19 associated with male factor infertility and oligoasthenoteratozoospermia. Hum. Reprod. 38, 1399–1411. doi: 10.1093/humrep/dead125. PMID: 37192818

[B30] Wu J. Lyu S. Guo D. Yang N. Liu Y. (2024). Protective effects of YCHD on the autoimmune hepatitis mice model induced by Ad-CYP2D6 through modulating the Th1/Treg ratio and intestinal flora. Front. Immunol. 15, 1488125. doi: 10.3389/fimmu.2024.1488125. PMID: 39606230 PMC11600021

[B29] Yu J. Sanjay Jaiswal V. Jang Y. Park M. Lee H. J. (2025). Salvia miltiorrhiza activates Nrf2/HO-1 signaling and restores steroidogenesis in Leydig TM3 cells and an aging rat model. Biomed. Pharmacother. 189, 118297. doi: 10.1016/j.biopha.2025.118297. PMID: 40582100

[B28] Zhang Y. Yang A. Zhao Z. Chen F. Yan X. Han Y. . (2024). Protein disulfide isomerase is essential for spermatogenesis in mice. JCI Insight 9, e177743. doi: 10.1172/jci.insight.177743. PMID: 38912589 PMC11383184

[B17] Zheng Y. Li D. M. Jiang X. H. Bai H. Z. Zhao G. C. (2024). A prediction model of sperm retrieval in males with idiopathic non-obstructive azoospermia for microdissection testicular sperm extraction. Reprod. Sci. 31, 366–374. doi: 10.1007/s43032-023-01362-1. PMID: 37749447

[B25] Zhou J. Hu S. Ouyang Y. Kong Y. Liu M. (2025). Immunometabolism and male reproductive function: linking inflammation, oxidative stress, and declining fertility. Front. Immunol. 16, 1736492. doi: 10.3389/fimmu.2025.1736492. PMID: 41445731 PMC12722913

