# Supplementary figures and images for: Gut Bacteroidales and AMH/INH-B ratio predict sperm retrieval: mechanistic insights via SCFA-mediated regulation of blood-testis barrier and steroidogenesis
Source: Front Cell Infect Microbiol. 2026 May 22;16:1777930. doi: 10.3389/fcimb.2026.1777930 (PMC13236950; doi:10.3389/fcimb.2026.1777930)

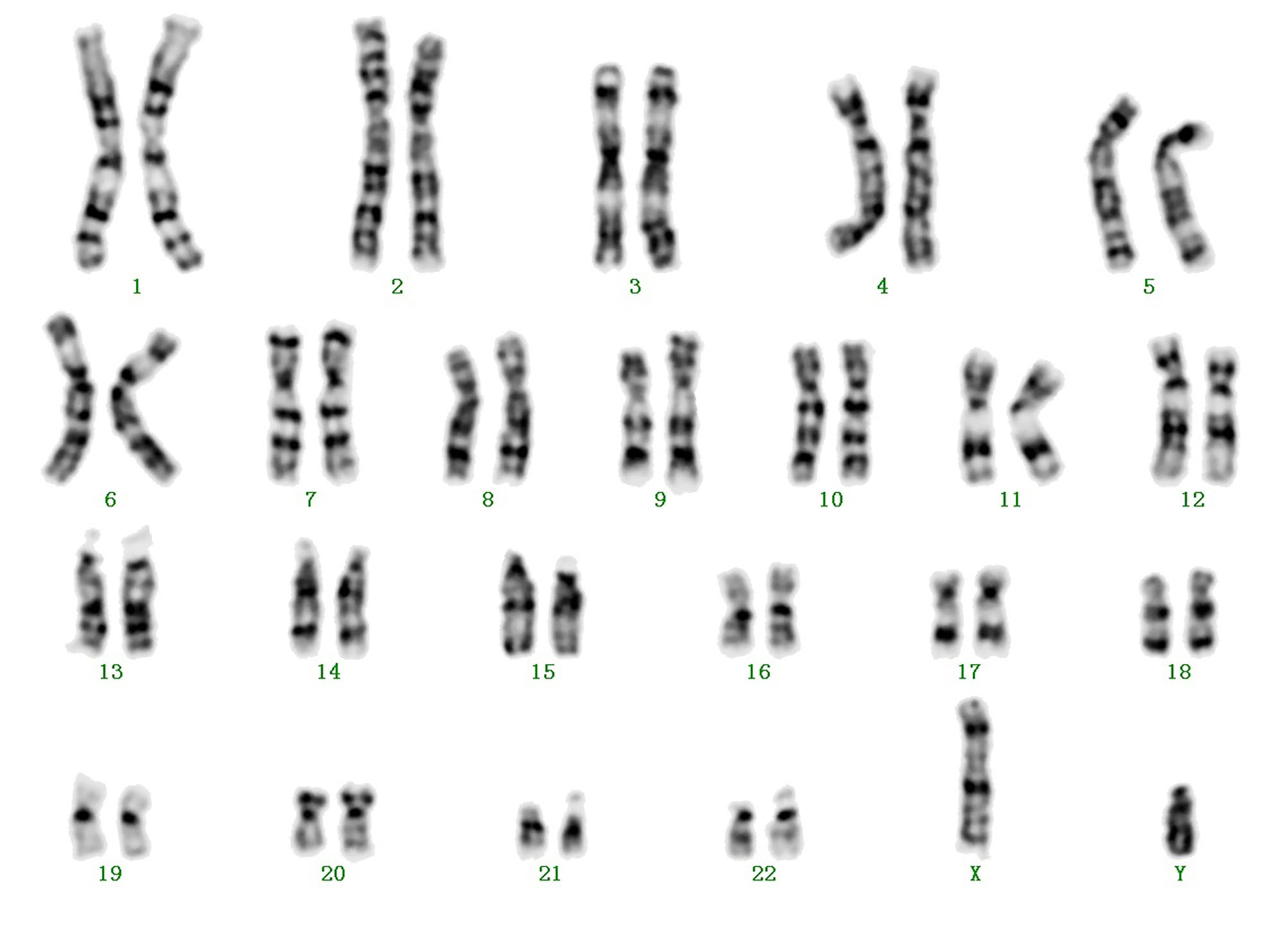

Supplement: Supplementary Figure 1 — Normal Karyotype of an FSH-normal NOA patient. [file Image1.jpeg]

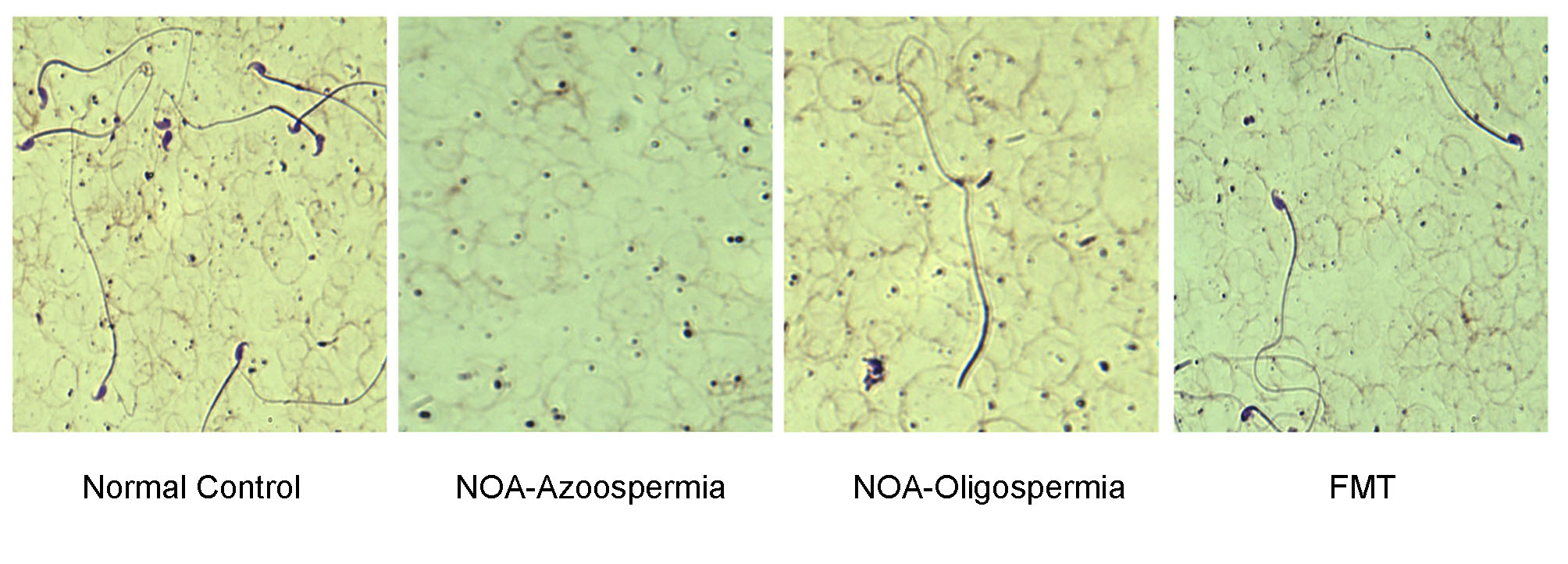

Supplement: Supplementary Figure 2 — Sperm morphology in different experimental groups. [file Image2.jpeg]

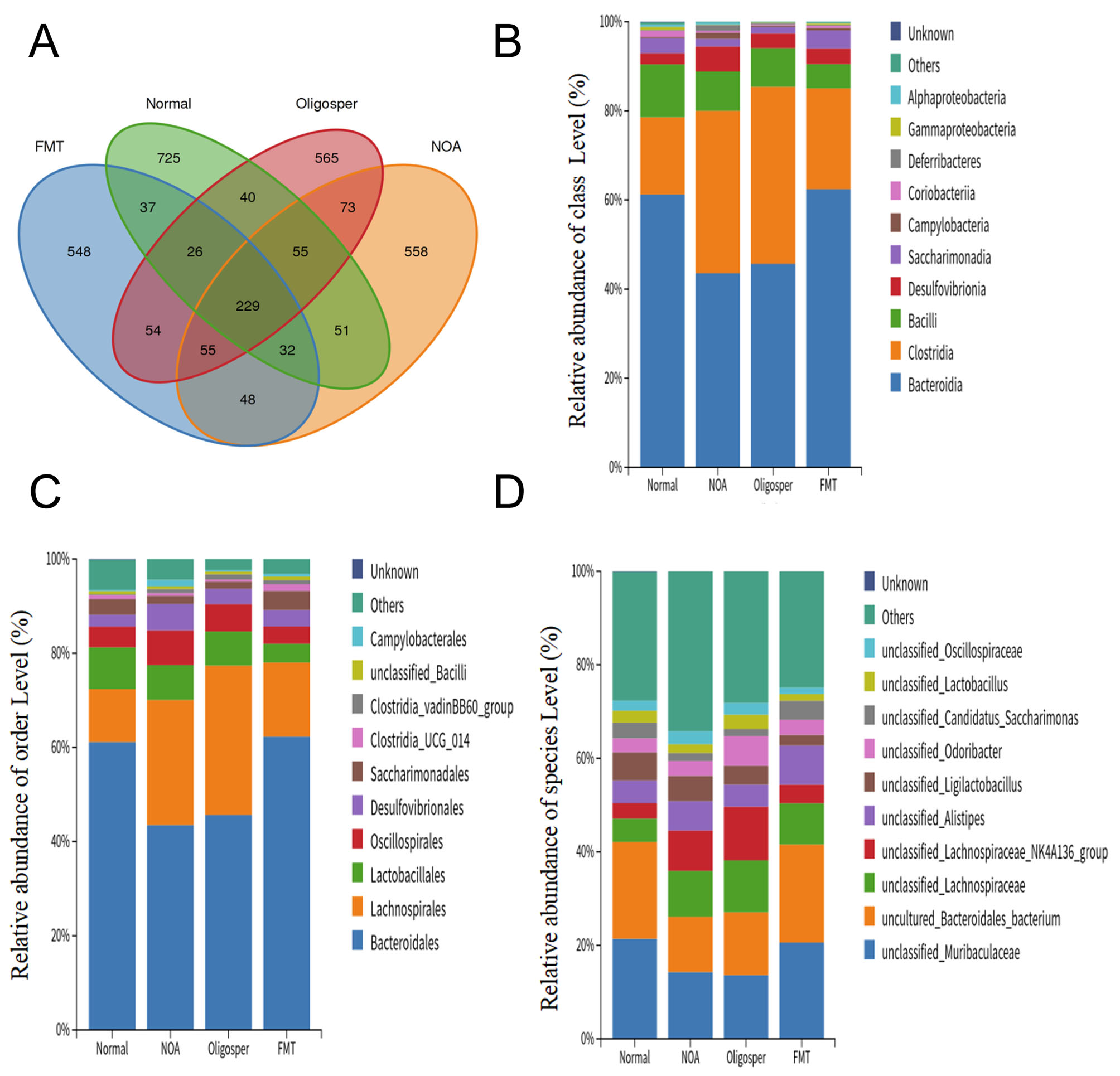

Supplement: Supplementary Figure 3 — Detailed analysis of gut microbiota dysbiosis and its reversal by FMT. (A) Veen analysis chart; (B-D) Relative abundance of class level (B), order level (C) and species level (D). [file Image3.jpeg]
